# Andrographolide Alleviates *Mycoplasma pneumoniae* Pneumonia in Children by Inhibiting Alveolar Epithelial Cell Pyroptosis Through the HMGB1/TLR4/NF-κB Pathway

**DOI:** 10.5812/ijpr-169826

**Published:** 2026-05-19

**Authors:** Lijuan Zhang, Umar Saeed, Rui Gao

**Affiliations:** 1 Department of Respiratory, Harbin 242 Hospital, Harbin, China; 2 Szechenyi Istvan University, Gyor, Hungary; 3 Korea University College of Health Sciences, Korea University, Seoul, South Korea; 4 Department of Respiratory Medicine, Xi'an Trade Union Hospital, Xi'an, China

**Keywords:** Pyroptosis, Andrographolide, Inflammation, Mycoplasma Pneumoniae Pneumonia, HMGB1/TLR4/NF-κB Pathway

## Abstract

**Background:**

*Mycoplasma pneumoniae* pneumonia (MPP) is a pulmonary inflammatory disease caused by *Mycoplasma pneumoniae* (Mp) infection that primarily involves the bronchi, alveoli, and pulmonary interstitium. It is the most common cause of community-acquired pneumonia in children. Andrographolide (AG) is a natural diterpenoid lactone with anti-inflammatory and immunomodulatory activities; however, its specific role and mechanism in MPP remain unclear.

**Objectives:**

This study aimed to investigate the mechanism by which AG inhibits pyroptosis in alveolar epithelial cells via the HMGB1/TLR4/NF-κB signaling pathway in the treatment of MPP in children.

**Methods:**

Mp-stimulated MLE-12 cells were treated with AG at 10, 20, or 50 μM. Cell viability and injury were assessed using CCK-8 and LDH assays, respectively. Protein expression levels of NLRP3, HMGB1, TLR4, and p-NF-κB p65 were determined by Western blotting, and cytokine levels (IL-6 and TNF-α) were measured by ELISA. In an Mp-infected mouse model, mice received AG at 25 or 50 mg/kg. Body weight, lung index, lung histopathology, pathway-related protein expression, and cytokine levels (IL-1β and TNF-α) in bronchoalveolar lavage fluid (BALF) were evaluated.

**Results:**

Mp significantly induced cytotoxicity, pyroptosis, and inflammation in vitro and in vivo (all P < 0.001). Andrographolide treatment dose-dependently reduced LDH release, suppressed HMGB1/TLR4/NF-κB/NLRP3 activation, and decreased proinflammatory cytokine levels (P < 0.05). In mice, AG improved survival-related metrics, ameliorated lung pathology, and inhibited pathway activity and cytokine secretion.

**Conclusions:**

Andrographolide mitigates MPP via the HMGB1/TLR4/NF-κB signaling pathway by inhibiting pulmonary epithelial cell pyroptosis and inflammation. These findings indicate its potential as a therapeutic agent.

## 1. Background

*Mycoplasma pneumoniae* pneumonia (MPP) is pulmonary inflammation caused by *Mycoplasma pneumoniae* (Mp) infection. As one of the smallest self-replicating prokaryotes, Mp lacks a cell wall, rendering it resistant to β-lactam antibiotics (1, 2). Mp accounts for approximately 10% - 40% of pneumonia cases and is particularly common in children (3, 4). In recent years, with the emergence and spread of drug-resistant strains, the therapeutic efficacy of macrolides has gradually declined, and the incidence of refractory *Mycoplasma pneumoniae* pneumonia (RMPP) has increased. This condition is characterized by persistent fever, severe pulmonary inflammation, and pulmonary and extrapulmonary complications, and it may lead to chronic sequelae, such as obliterative bronchiolitis and atelectasis (4-6). Therefore, there is an urgent need to elucidate the molecular mechanisms of Mp pathogenesis and develop new therapeutic strategies targeting the host inflammatory response.

The pathogenicity of Mp primarily depends on adhesion to the respiratory epithelium and the production of virulence factors. Surface adhesins, such as the P1 and P30 proteins, bind to glycoprotein receptors, enabling colonization (7, 8). In addition, the community-acquired respiratory distress syndrome toxin (CARDS Tx) secreted by Mp can cause vacuolization of airway cells and strongly activate the NLRP3 inflammasome (9, 10). Recent studies have shown that Mp infection can induce pyroptosis in alveolar epithelial cells, a programmed inflammatory form of cell death. This process depends on inflammasome activation, caspase-1 activation, and gasdermin D (GSDMD) cleavage, ultimately leading to cell membrane pore formation and the release of large amounts of proinflammatory cytokines, such as IL-1β and IL-18 (11-13). In the immune signaling network triggered by Mp, high mobility group box 1 (HMGB1) is a key damage-associated molecular pattern (DAMP) that can be actively secreted by necrotic or immune cells (14, 15). After binding to Toll-like receptor 4 (TLR4), extracellular HMGB1 activates nuclear factor κB (NF-κB) through a myeloid differentiation factor 88 (MyD88)-dependent pathway. Activated NF-κB then translocates to the nucleus and initiates the transcription of multiple inflammatory factor genes, such as TNF-α, IL-6, and IL-1β, forming a positive feedback loop that amplifies the inflammatory response (16, 17). Meanwhile, Mp and its toxins can also directly or indirectly activate the NLRP3 inflammasome through signaling events such as potassium ion efflux, reactive oxygen species (ROS) production, and lysosomal rupture (18, 19). Moreover, caspase-1 activated by the NLRP3 inflammasome not only cleaves GSDMD to induce pyroptosis but also promotes the maturation and secretion of IL-1β and IL-18, further driving neutrophil infiltration and tissue damage (20, 21).

Notably, studies have shown close interactions between the HMGB1/TLR4/NF-κB signaling axis and NLRP3 inflammasome activation (22, 23). The NF-κB pathway provides priming signals for NLRP3 and pro-IL-1β expression, and HMGB1 can enhance NLRP3 oligomerization and activation (24, 25). This multifaceted positive feedback inflammatory regulatory network is likely to be a core mechanism underlying excessive inflammatory responses and tissue damage during Mp infection. Therefore, targeting and modulating this signaling axis to inhibit excessive DAMP-mediated innate immune activation may provide a novel therapeutic strategy for controlling the progression of Mp pneumonia.

## 2. Objectives

Andrographolide (AG), a natural diterpene lactone extracted from Andrographia paniculata, has been shown to possess various pharmacological activities, including anti-inflammatory, antioxidant, antiviral, and immunomodulatory effects (26, 27). Studies have shown that AG exerts anti-inflammatory effects through multiple mechanisms: it blocks the NF-κB signaling pathway by inhibiting IκBα degradation and p65 nuclear translocation, and it downregulates the phosphorylation levels of MAPK and Akt, thereby influencing inflammatory gene expression (28, 29). AG also reduces oxidative stress by scavenging ROS and enhancing antioxidant enzyme activity (30). Emerging evidence indicates that AG may suppress NLRP3 inflammasome activation and attenuate pyroptosis (31). However, whether AG can alleviate Mp-induced pyroptosis in alveolar epithelial cells and lung tissue inflammation by intervening in the key signaling network of HMGB1/TLR4/NF-κB/NLRP3 has not been systematically investigated in vitro or in vivo. Therefore, this study systematically evaluated the protective effects of AG on lung epithelial cell injury caused by Mp infection in vitro and in vivo and explored its molecular mechanisms, aiming to provide a theoretical basis and a potential drug-intervention strategy for the clinical treatment of drug-resistant Mp pneumonia.

## 3. Methods

### 3.1. Reagents and Compounds

Andrographolide (AG; purity ≥ 98%; Sigma) was dissolved in dimethyl sulfoxide (DMSO) to prepare a 50 mM stock solution. The final DMSO concentration did not exceed 0.1% in any experimental group. Antibodies against NLRP3, HMGB1, phosphorylated NF-κB p65, NF-κB p65, TLR4, and β-actin were obtained from Thermo Fisher Scientific, Abcam, Santa Cruz Biotechnology, and Proteintech. All ELISA kits were obtained from MultiSciences Biotech Co., Ltd. The CCK-8 and LDH assay kits were supplied by Dojindo and Beyotime, respectively. Reagents for qPCR, including TRIzol, PrimeScript RT reagent kit, and SYBR Premix Ex Taq II, were obtained from Invitrogen and Takara, and primers were synthesized by Sangon Biotech (Shanghai).

### 3.2. Cell Culture

Mouse alveolar epithelial cells (MLE-12; ATCC) were cultured in DMEM containing 10% fetal bovine serum and 1% penicillin/streptomycin at 37 °C in a 5% CO_2_ atmosphere. *Mycoplasma pneumoniae* (Mp; FH strain; ATCC 15531) was cultured in PPLO broth medium to the logarithmic growth phase, collected by centrifugation, and resuspended in PBS. The bacterial concentration was adjusted to 1 × 10^9^ CCU/mL. For the infection model, cells were incubated with Mp at a multiplicity of infection (MOI) of 10 for 24 hours. Cells were divided into the following groups: control, Mp-infected, and Mp-infected plus AG at 10, 20, or 50 μM. All in vitro experiments were performed in triplicate and repeated independently at least 3 times.

### 3.3. Animals and In Vivo Model

Female Balb/c mice aged 4 - 6 weeks and weighing 18 - 22 g were housed in a standard animal center at 22 ± 2 °C, 50% ± 10% humidity, and under a 12-hour light/dark cycle. After 1 week of acclimatization, the mice were randomly divided into 4 groups (n = 10 per group): control group, Mp group, and Mp + AG groups at 25 and 50 mg/kg.

*Mycoplasma pneumoniae* (Mp) was collected, and the concentration was adjusted to 2 × 10^8^ CCU/mL. The bacterial suspension was slowly instilled into both nostrils of the mice (25 μL per nostril). Mice in the control group received 25 μL of PBS per nostril. Starting on the second day after infection, AG at 25 or 50 mg/kg, dissolved in 0.5% CMC-Na solution, was administered once daily for 7 consecutive days. The control and Mp model groups received an equivalent volume of 0.5% CMC-Na solution. Body weight was monitored daily during this period. At the end of the experiment, 1% pentobarbital sodium (50 mg/kg) was injected intraperitoneally to anesthetize the mice. Blood was collected from the eyeball, and serum was separated and stored for ELISA detection. Whole lung tissues were collected, rinsed with PBS, blotted dry with filter paper, and weighed. Some tissues were fixed in 4% paraformaldehyde for pathological examination. The remaining tissues were preserved in liquid nitrogen for subsequent Western blot and qPCR analyses.

### 3.4. Cell Viability and Cytotoxicity Assays

Cell viability was assessed using the CCK-8 method. MLE-12 cells were seeded in 96-well plates at 1 × 10^4^ cells/well. After 24 hours of culture, cells were treated according to the groups described above. After treatment, 10 μL of CCK-8 solution was added to each well, and the cells were incubated at 37 °C in the dark for 1.5 hours. Absorbance (OD) was measured at 450 nm using an enzyme-linked immunosorbent assay reader, and the cell survival rate and inhibition rate were calculated.

### 3.5. Detection of Lactate Dehydrogenase Release

Cell supernatants were collected and processed according to the instructions of the LDH kit (Beyotime, C0017). Absorbance was measured at 490 nm to assess the extent of cell membrane damage.

### 3.6. Protein Expression Analysis by Western Blotting

Total protein was extracted from each group of cells or lung tissues using RIPA lysis buffer containing protease and phosphatase inhibitors and was quantified using the BCA method. Protein samples (30 μg) were separated by 10% SDS-PAGE and transferred to a PVDF membrane. The membrane was blocked with 5% skimmed milk for 2 hours at room temperature, incubated with primary antibodies overnight at 4 °C, washed with TBST, and then incubated with an HRP-labeled goat anti-rabbit/mouse secondary antibody for 1 hour at room temperature. Images were developed using an ECL chemiluminescence kit, and gray values were analyzed using Image Lab software (Bio-Rad). Protein expression levels were normalized to β-actin.

### 3.7. Cytokine Measurements

According to the kit instructions, levels of TNF-α, IL-6, and IL-1β in cell culture supernatants, mouse serum, and BALF were measured using ELISA kits. Absorbance was read at 450 nm, and cytokine concentrations were calculated based on standard curves.

### 3.8. Quantitative Real-Time PCR

Total RNA was extracted from lung tissue using TRIzol reagent, and purity and concentration were determined. Total RNA (1 μg) was reverse-transcribed according to the kit instructions. qPCR was performed using SYBR Premix Ex Taq II on the CFX96 Touch system. The 20-μL reaction system consisted of 10 μL of SYBR Premix, 2 μL of cDNA, 0.8 μL each of upstream and downstream primers, and 6.4 μL of ddH_2_O. The reaction conditions were 95 °C for 30 seconds, followed by 40 cycles of 95 °C for 5 seconds and 60 °C for 30 seconds. Using GAPDH as the internal reference, relative mRNA expression was calculated using the 2^-ΔΔCt^ method. The primer sequences are listed in Table 1.

**Table 1.** Gene Primer Sequence

Gene and Primer sequences	Length (bp)
**TLR4**	121
GGACTCTGATCATGGCACTG	
CTGATCCATGCATTGGTAGGT	
**NF-κB p65**	149
CAGCCAAAGAAGGACACGAC	
TGGTGGTACTGCTTGGTCTTC	
**NLRP3**	115
AGACCTCCGCGAGAAACTG	
CGTGCATTATCTGAACCCCAC	
**GAPDH**	123
AGGTCGGTGTGAACGGATTTG	
TGTAGACCATGTAGTTGAGGTCA	

### 3.9. Histopathological Evaluation

Lung tissue was fixed in 4% paraformaldehyde and embedded in paraffin to prepare 4-μm-thick sections. The tissue was processed according to the instructions of the hematoxylin and eosin (H&E) staining kit, including xylene dewaxing, graded alcohol hydration, hematoxylin staining of nuclei, eosin staining of cytoplasm, and sealing with neutral gum. Pathological changes in lung tissue, including alveolar structure, inflammatory cell infiltration, hemorrhage, and edema, were observed under an optical microscope, and semi-quantitative analysis was conducted based on pre-established scoring criteria.

### 3.10. Statistical Analysis

All data are expressed as mean ± standard error of the mean (SEM). Statistical analyses were performed using SPSS 23.0 software. One-way ANOVA followed by the LSD post hoc test was used for multiple comparisons. A P value < 0.05 was considered statistically significant.

## 4. Results

### 4.1. Andrographolide Restores Viability and Reduces Cytotoxicity in Mp-Infected Cells

**Figure 1.**
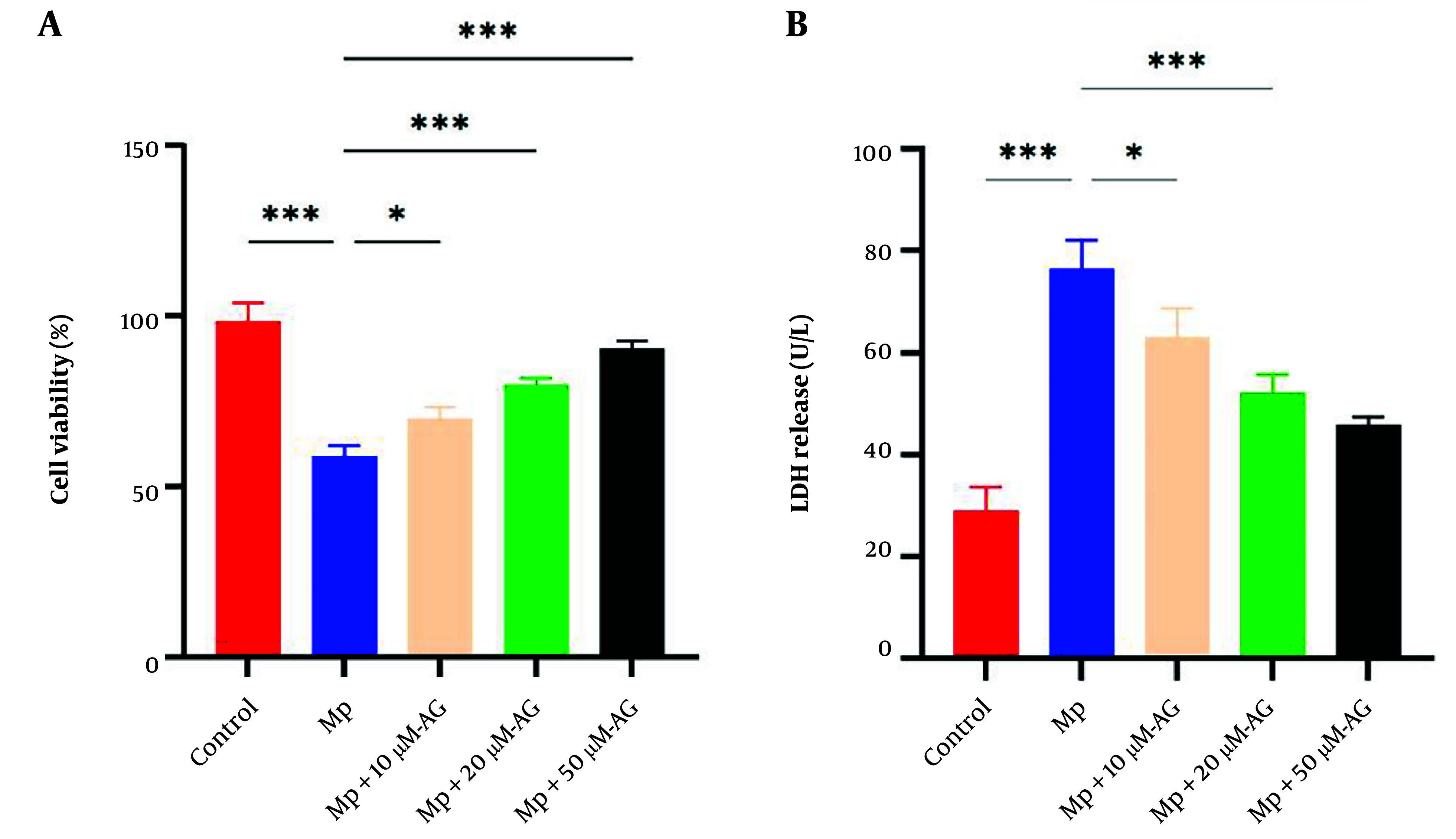
Effects of andrographolide (AG) on cell viability and LDH release in Mp-infected MLE-12 cells. A, Cell viability of MLE-12 cells; B, LDH release in MLE-12 cells [data are expressed as mean ± SEM (N = 3); *P < 0.05, **P < 0.01, ***P < 0.001].

We evaluated the effects of AG on MLE-12 cell function after *Mycoplasma pneumoniae* infection by assessing proliferation activity and membrane integrity. The results (Figure 1A) showed that, compared with the control group, Mp infection significantly inhibited MLE-12 cell proliferation, whereas treatment with different concentrations of AG (10, 20, and 50 μM) significantly increased cell survival rates, demonstrating a concentration-dependent restorative effect (P < 0.05).

The LDH release results shown in Figure 1B indicated that Mp infection significantly increased cytotoxicity, whereas AG intervention significantly reduced LDH release (P < 0.05), indicating that AG effectively protected cell membrane integrity (P < 0.05).

### 4.2. Andrographolide Suppresses NLRP3 Inflammasome Activation

**Figure 2.**
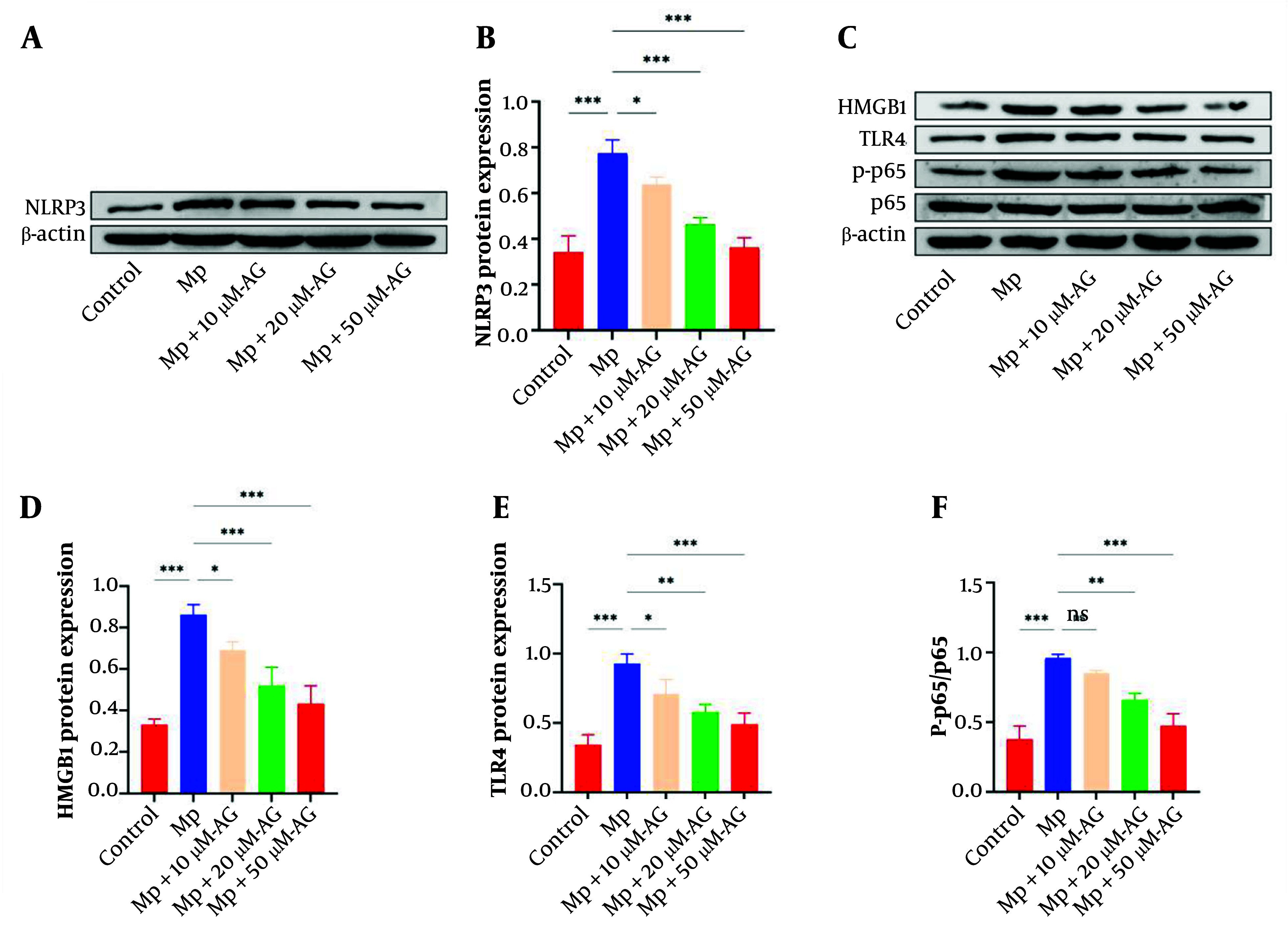
Effects of AG on NLRP3 and HMGB1/TLR4/NF-κB pathway protein expression in Mp-infected MLE-12 cells. A, Representative Western blot bands of NLRP3 protein; B, Quantitative analysis of NLRP3 protein expression; C, Representative Western blot bands of HMGB1, TLR4, phosphorylated p65 (p-p65), and total p65; D-F, Quantitative analysis of protein expression levels [data are expressed as mean ± SEM (N = 3); *P < 0.05, **P < 0.01, ***P < 0.001].

NLRP3 is a core component of the inflammasome, and its activation can induce pyroptosis. We assessed the expression of NLRP3, a key signaling molecule for pyroptosis, by Western blotting. As shown in Figure 2A, Mp infection significantly upregulated NLRP3 expression (P < 0.001), whereas AG treatment at 10 - 50 μM dose-dependently inhibited this increase (P < 0.05), indicating that AG alleviated Mp-induced pyroptosis (Figure 2B).

### 4.3. Andrographolide Inhibits HMGB1/TLR4/NF-κB Signaling Activation

We evaluated the effects of AG on the HMGB1/TLR4/NF-κB inflammatory signaling axis by Western blotting. The results showed (Figure 2C) that Mp infection significantly increased the protein levels of HMGB1 and TLR4 and the phosphorylation level of p65 (P < 0.001). AG treatment at 10 - 50 μM significantly downregulated the expression of HMGB1 and TLR4, and medium and high concentrations of AG (20 and 50 μM) significantly inhibited p65 phosphorylation (P < 0.01) (Figure 2D-F). These results suggest that AG may exert anti-inflammatory effects by inhibiting this signaling pathway through multiple targets.

### 4.4. Andrographolide Attenuates Inflammatory Cytokine Secretion

**Figure 3.**
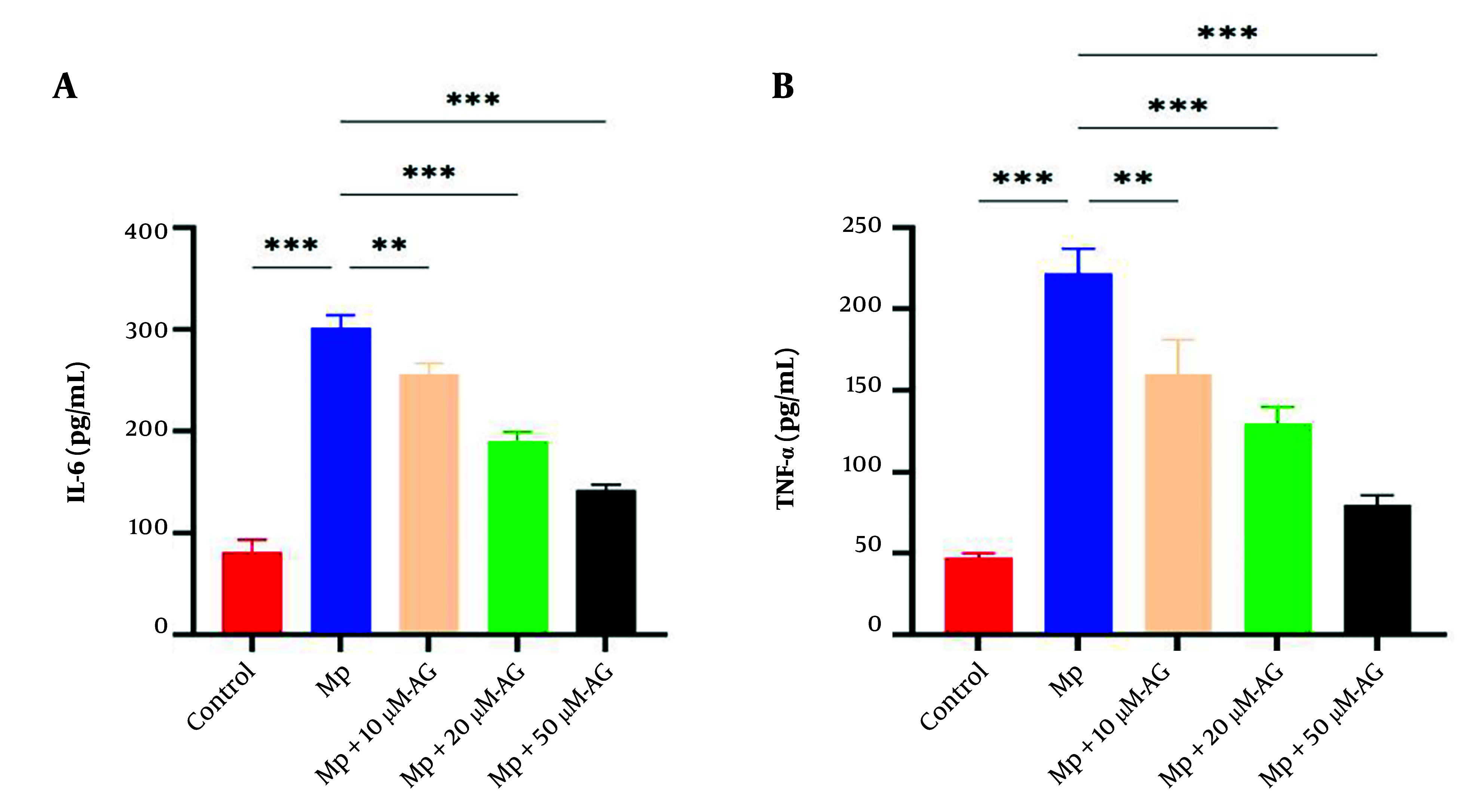
Effects of AG on the levels of inflammatory cytokines in the supernatant of Mp-infected MLE-12 cells. A, IL-6 level; B, TNF-α level [data are expressed as mean ± SEM (N = 3); *P < 0.05, **P < 0.01, ***P < 0.001].

We also assessed the effects of AG on the levels of the inflammatory cytokines IL-6 and TNF-α in MLE-12 cells by ELISA. The results showed (Figure 3) that the concentrations of IL-6 and TNF-α significantly increased after Mp infection (P < 0.001). After AG intervention, the release of these two inflammatory factors was significantly reduced (P < 0.01), with the strongest inhibitory effect at 50 μM, further confirming the anti-inflammatory effect of AG.

### 4.5. Andrographolide Improves Systemic Manifestations and Lung Edema in Mice

**Figure 4.**
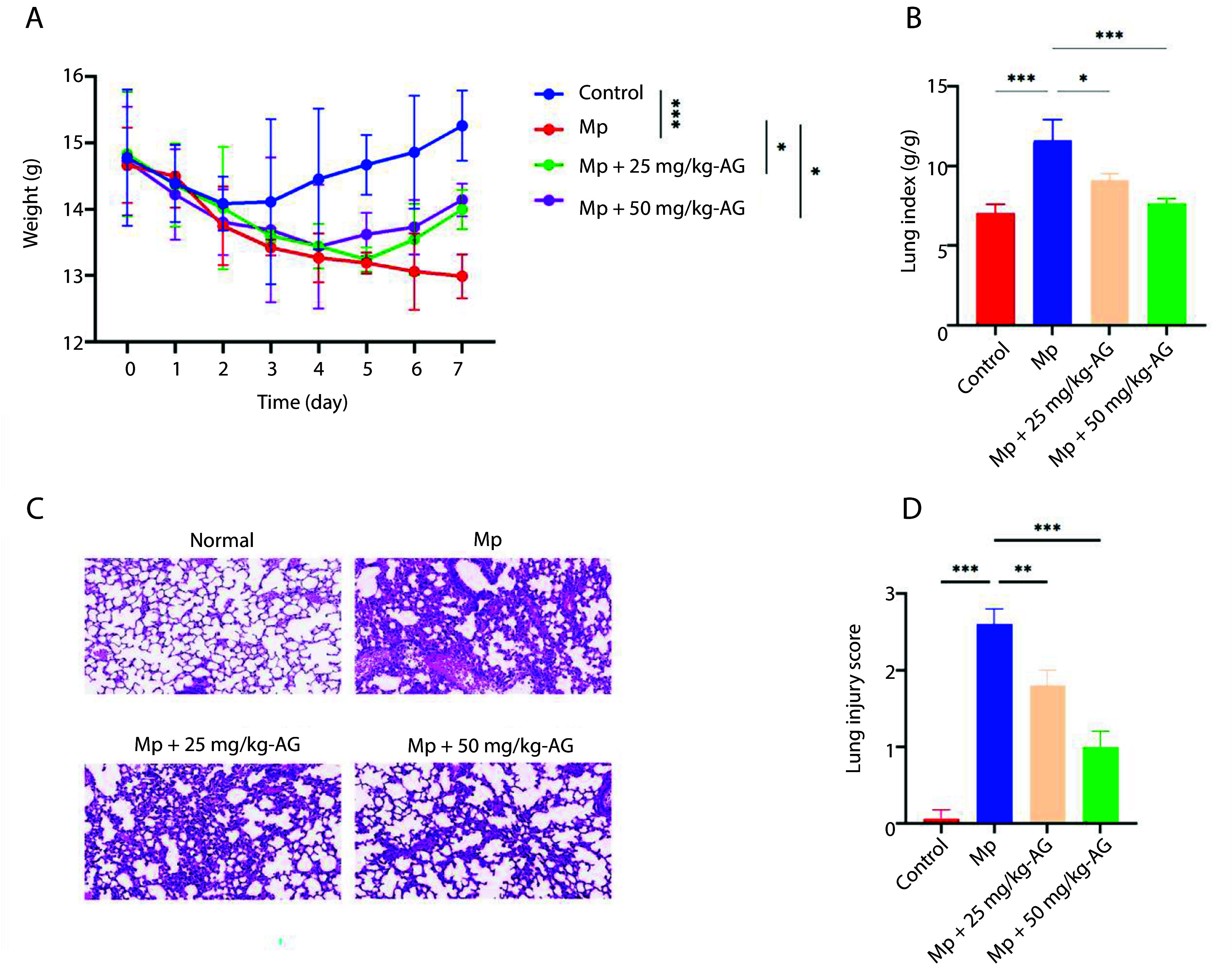
Effects of AG on general condition and lung histopathology in Mp-infected mice. A, Representative images of H&E-stained lung tissue sections (scale: 100 μm); B, Quantitative scoring results of pathological lung injury; C, Representative images of H&E-stained lung tissue sections (scale: 100 μm); D, Quantitative scoring results of pathological lung injury [data are expressed as mean ± SEM (A-B: N = 10; C-D: N = 3); *P < 0.05, **P < 0.01, ***P < 0.001].

The in vivo results showed that Mp infection led to a significant decrease in mouse body weight (Figure 4A) and a significant increase in lung index (lung weight/body weight) (Figure 4B), indicating disease activity and lung tissue edema (P < 0.001). However, AG treatment at 25 and 50 mg/kg effectively reversed weight loss and reduced the lung index (P < 0.05), indicating that AG has a protective effect against Mp infection.

### 4.6. Andrographolide Alleviates Lung Histopathological Injury

H&E staining of lung tissue (Figure 4C) showed that AG treatment significantly alleviated the severe destruction of alveolar structure, inflammatory cell infiltration, and interstitial thickening caused by Mp infection. The semi-quantitative lung injury scores (Figure 4D) indicated that the pathological changes caused by Mp infection were significantly alleviated after AG treatment, particularly at the higher dose of 50 mg/kg (P < 0.01). The H&E staining results further demonstrate that AG could serve as a potential therapeutic agent for *Mycoplasma pneumoniae* pneumonia.

### 4.7. Andrographolide Suppresses the Pulmonary HMGB1/TLR4/NF-κB Pathway In Vivo

**Figure 5.**
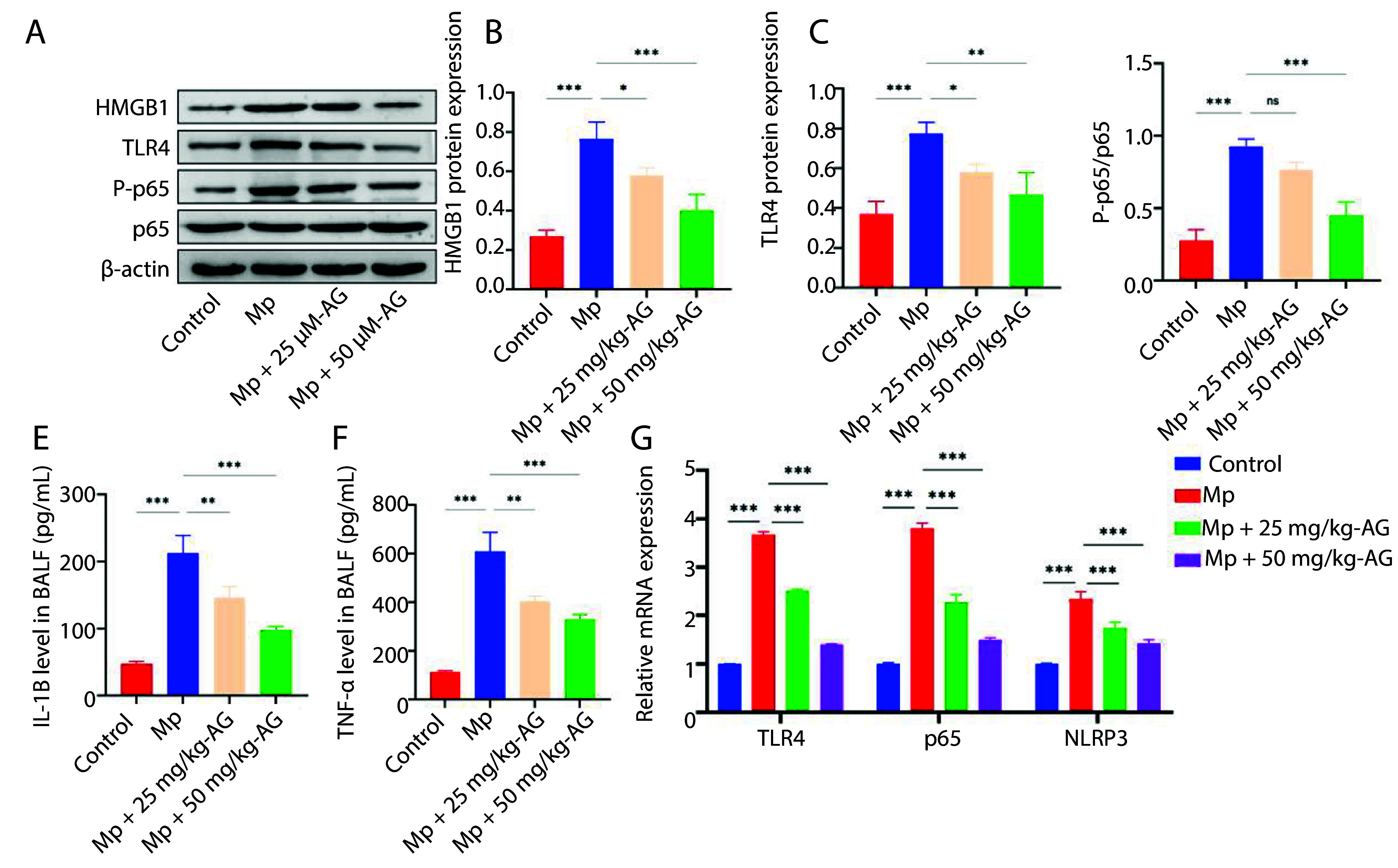
Effects of AG on the HMGB1/TLR4/NF-κB pathway, inflammatory cytokines, and gene expression in Mp-infected mice. (A) Representative Western blot bands of HMGB1, TLR4, p-p65, and total p65 in lung tissues. (B-D) Quantitative analysis of protein expression levels. (E) IL-1β level in BALF detected by ELISA. (F) TNF-α level in BALF detected by ELISA. (G) Effects of AG on the mRNA expression of inflammation-related genes in lung tissues of Mp-infected mice. mRNA expression levels of TLR4, NF-κB p65, and NLRP3 were detected by qPCR [data are expressed as mean ± SEM (N = 3); *P < 0.05, **P < 0.01, ***P < 0.001].

We analyzed the expression of proteins in the inflammatory signaling pathway in lung tissue (Figure 5A). The results (Figure 5B-D) showed that Mp infection significantly upregulated the expression of HMGB1, TLR4, and p-p65 in lung tissue (P < 0.001), indicating that Mp activated the inflammatory response in lung tissue. Treatment with AG at 25 and 50 mg/kg reversed these changes. The expression levels of HMGB1, TLR4, and p-p65 proteins in the AG treatment groups were significantly inhibited, and the inhibitory effect of AG was dose-dependent, with the high dose of AG (50 mg/kg) showing the most pronounced effect (P < 0.05).

### 4.8. Andrographolide Reduces Inflammatory Cytokines in BALF

As shown in Figure 5E-F, to further quantify the pulmonary inflammatory response, we measured the levels of the key proinflammatory cytokines IL-1β and TNF-α in BALF. Compared with the control group, the concentrations of IL-1β and TNF-α in the Mp infection model group were significantly increased (P < 0.001), indicating robust pulmonary inflammation triggered by Mp infection. After AG intervention, this abnormal inflammatory state was effectively controlled. All AG treatment groups (25 and 50 mg/kg) showed significantly reduced levels of IL-1β and TNF-α in BALF (P < 0.01), indicating that AG has a clear anti-inflammatory effect. These findings further confirmed, at the cytokine level, that AG can effectively inhibit the excessive inflammatory response driven by Mp infection, providing an important basis for its potential mechanism in treating *Mycoplasma pneumoniae* pneumonia.

### 4.9. Andrographolide Downregulates Inflammation-Related Gene Expression

To further explore the anti-inflammatory mechanism of AG at the transcriptional level, we used qPCR to measure the mRNA expression of key inflammatory signaling molecules in lung tissue. The results are shown in Figure 5G. Mp infection significantly upregulated the gene expression levels of TLR4, NF-κB p65, and NLRP3 (P < 0.001). After AG treatment, the abnormally high expression of these genes was significantly inhibited (P < 0.001). This result confirmed that AG can effectively inhibit the TLR4/NF-κB/NLRP3 inflammatory signaling pathway activated by Mp infection at the transcriptional level.

## 5. Discussion

*Mycoplasma pneumoniae* is a leading cause of community-acquired pneumonia in children, and its pathogenesis is fundamentally linked to an excessive host inflammatory response rather than direct bacterial toxicity (32). A key driver of this immunopathology is activation of the NLRP3 inflammasome, which triggers pyroptosis, a highly inflammatory form of programmed cell death, and the release of potent proinflammatory cytokines (33). Our study provides novel evidence that AG, a natural diterpenoid lactone, confers significant protection against Mp-induced pneumonia by targeting the HMGB1/TLR4/NF-κB signaling axis, thereby suppressing NLRP3 inflammasome-mediated pyroptosis and the subsequent cytokine storm.

The pivotal role of the NLRP3 inflammasome in Mp infection has been previously established. Segovia et al. demonstrated that Mp and its CARDS toxin activate the NLRP3/ASC/caspase-1 inflammasome complex in macrophages, which is essential for IL-1β processing and secretion, as well as for an effective innate immune response and bacterial clearance in mice (35). Our findings align with this concept, confirming that Mp infection significantly upregulates NLRP3 expression both in cultured alveolar epithelial cells and in lung tissue. More importantly, we extend this understanding by identifying upstream regulators, namely the DAMP molecule HMGB1 and its receptor TLR4. Extracellular HMGB1, a classic DAMP released during cell stress or necrosis, acts as a late mediator of inflammation (35). Its interaction with TLR4 initiates a MyD88-dependent signaling cascade leading to activation of the transcription factor NF-κB, which in turn promotes the expression of NLRP3 and pro-IL-1β (36). Our data clearly demonstrate that Mp infection triggers HMGB1 release and upregulates TLR4 expression, resulting in NF-κB activation. Andrographolide treatment effectively interrupts this upstream signaling, leading to downregulation of NLRP3 expression. This mechanism is strongly supported by recent studies in various disease models. For instance, in acetaminophen-induced hepatotoxicity, scopolin was shown to exert protective effects by concurrently modulating the Nrf2 antioxidant pathway and inhibiting the HMGB1/TLR4/NF-κB/NLRP3 axis (37). Similarly, tetramethylpyrazine alleviated LPS-induced acute lung injury by specifically inhibiting the HMGB1/TLR4/NF-κB pathway (38). Most notably, in the context of ischemia/reperfusion injury, panaxynol directly improved cardiac function by inhibiting NLRP3-induced pyroptosis and apoptosis via the HMGB1/TLR4/NF-κB axis (39). Our study is the first to integrate this mechanistic framework into the context of Mp pneumonia. We demonstrate that AG, by targeting the HMGB1/TLR4/NF-κB axis, effectively suppresses subsequent NLRP3 activation and pyroptosis in alveolar epithelial cells, the primary target cells in Mp infection. This effect was consistent across all our models, as evidenced by reduced LDH release and decreased cleaved caspase-1 expression in vitro, alongside significant amelioration of lung histopathology and decreased levels of the inflammatory cytokines IL-1β, TNF-α, and IL-6 in vivo.

The innovation of our work lies in systematically delineating the complete signaling pathway during the Mp pathological process, from the initial release of HMGB1 to recognition by TLR4, subsequent activation of NF-κB, activation of the NLRP3 inflammasome, and the terminal process of inflammatory cell death, namely pyroptosis, while demonstrating that AG acts across the entire pathway. This offers a therapeutic strategy that is more comprehensive and mechanistically grounded than approaches targeting single downstream components. Despite these compelling findings, certain limitations must be acknowledged. The direct molecular targets of AG within this pathway remain to be precisely identified. Furthermore, the potential synergistic effect of AG combined with conventional macrolide antibiotics against Mp was not explored in this study. Future investigations using tissue-specific knockout models and exploring combination therapies will be valuable for validating this mechanism and advancing AG toward potential clinical application.

## 5.1. Conclusions

Our findings indicate that AG alleviates Mp-induced lung injury by disrupting the HMGB1/TLR4/NF-κB signaling pathway, thereby inhibiting NLRP3 inflammasome activation and pyroptosis in alveolar epithelial cells. These findings not only deepen our understanding of the immunopathology of Mp infection but also position AG as a promising candidate for host-directed therapeutic approaches, particularly for alleviating the excessive inflammation associated with refractory or antibiotic-resistant Mp pneumonia.

In addition, this study showed that andrographolide effectively improved Mp-induced pneumonia in vivo and in vitro. Andrographolide alleviated Mp-induced alveolar epithelial cell pyroptosis and LDH release, reduced NLRP3 inflammasome activation, and decreased the release of inflammatory cytokines such as IL-6 and TNF-α. Furthermore, AG significantly improved Mp-induced weight loss, pulmonary edema, and histopathological damage in lung tissue. Andrographolide also downregulated the expression of HMGB1, TLR4, and p-p65, as well as the levels of inflammatory factors such as TLR4, NF-κB p65, and NLRP3. Taken together, our results indicate that AG exerts its anti-inflammatory effects primarily by targeting the HMGB1/TLR4/NF-κB/NLRP3 axis, suggesting its potential therapeutic value for Mp pneumonia.

## Data Availability

The datasets generated and analyzed during the current study are available from the corresponding author upon reasonable request. The raw data are not publicly stored in a repository due to the nature of the original experimental records and animal model data.
